# Morphological Reconstruction for Variable Wing Leading Edge Based on the Node Curvature Vectors

**DOI:** 10.3390/biomimetics9040250

**Published:** 2024-04-20

**Authors:** Jie Zeng, Qingfeng Zhu, Yueqi Zhao, Zhigang Wang, Yu Yang, Qi Wu, Jinpeng Cui

**Affiliations:** 1 State Key Laboratory of Mechanics and Control of Mechanical Structures, Nanjing University of Aeronautics and Astronautics, Nanjing 210016, China; qing-feng@nuaa.edu.cn (Q.Z.); sxyueqizhao@nuaa.edu.cn (Y.Z.); sz2301089@nuaa.edu.cn (J.C.); 2 Aircraft Strength Research Institute of China, National Key Laboratory of Strength and Structural Integrity, Xi’an 710065, China; wangzhigang@cae.edu.cn (Z.W.); yangy076@avic.com (Y.Y.); wuqi@hnu.edu.cn (Q.W.); 3 Department of Aeronautical Science and Engineering, Beihang University, Beijing 100191, China

**Keywords:** morphological reconstruction, variable wing leading edge, node curvature vectors, curvature propagation method, strain–curvature function

## Abstract

Precise morphology acquisition for the variable wing leading edge is essential for its bio-inspired adaptive control. Therefore, this study proposes a morphological reconstruction method for the variable wing leading edge, utilizing the node curvature vectors-based curvature propagation method (NCV-CPM). By establishing a strain–arc curvature function, the method fundamentally mitigates the impact of surface curvature angle on curvature computation accuracy at sensing points. We introduce a technique that uses high-order curvature fitting functions to determine the curvature vectors of arc segment nodes. This method reduces cumulative errors in curvature computation linked to the linear interpolation-based curvature propagation method (LI-CPM) at unattached sensor positions. Integrating curvature–strain functions aids in wing leading-edge strain field reconstruction, supporting structural health monitoring. Additionally, a particle swarm algorithm optimizes the sensing point distribution, reducing network complexity. This study demonstrates significantly enhanced morphological reconstruction accuracy compared to those obtained with conventional LI-CPM.

## 1. Introduction

The design inspiration for morphing airfoils is derived from the wing structures of birds and other flying animals. It aims to achieve morphing capabilities in aircraft to optimize flight performance and fuel efficiency in different flight phases or conditions [1,2]. Concurrently, researchers are also dedicated to investigating adaptive control methods for variable wing structures to achieve enhanced maneuverability, stability, and energy efficiency. Chen et al. developed and successfully implemented control laws on a tensegrity morphing airfoil based on reduced order Class-k tensegrity dynamics [3]. Shen et al. proposed an optimal control method based on Markov parameters to achieve wing shape control for tensegrity morphing airfoils [4].

To achieve the “propagation-shifting” functionality of aircraft, ensuring their optimal aerodynamic profile, aerodynamic performance, and stealth characteristics in response to complex flight environments and mission requirements, it is essential to synchronize and close the loop control of the wing leading-edge propagation perception and airfoil adjustment [5,6].

Currently, conventional structural propagation perception technologies mainly include two types: non-contact and contact. Non-contact shape measurement technologies mainly include methods such as visual measurement and laser scanning [7,8]. Although this method has high measurement accuracy, it has visual blind spots and presents certain limitations in terms of volume size, installation calibration, vibration resistance, and real-time response characteristics.

Compared to non-contact measurement modes, deformation monitoring methods based on flexible, contact-type strain information perception are more suitable for the real-time shape identification of in-service aircraft structures. For example, the modal superposition method requires the prior acquisition of accurate load or material property information, that is, identifying the strain mode and displacement mode matrices of the target structure through a large number of experiments or finite element modeling. However, due to the complexity of the wing leading-edge structure and material properties, the engineering applicability of this method is limited [9]. For example, the global linear regression prediction or segmented continuous interpolation fitting method, despite having a simple reconstruction process, requires a large number of sensors to ensure the accuracy of structural shape reconstruction, limiting its practical application in the field of variable curvature wing leading-edge propagation perception [10].

To address the above problems, researchers have proposed a linear interpolation-based curvature propagation method (LI-CPM), which reconstructs structural displacement field information by establishing the mathematical relationship between strain, curvature, and displacement without requiring any prior knowledge of material properties or load size, and only using a finite number of discrete strain data [11,12].

Palma et al. obtained the curvature distribution characteristics along the thickness direction of a structure after loading and the position of the neutral layer of the strain by implanting multiple optical fiber sensors into a 3D composite material plate [13]. Li et al. achieved structural propagation perception by constructing a function relationship model between measured strain sensor data and beam structure curvature [14]. Cheng et al. studied a morphing reconstruction method for thin plates supported by multiple elastic supports based on the curvature–displacement function and the Runge–Kutta solving principle [15]. Liu et al. achieved the displacement field reconstruction of the bed of a gantry boring and milling machine under random loads using a continuous curvature function constructed using the cubic spline interpolation method [16]. Roesthuis et al. studied a method for calculating the curvature of a driving beam based on the measured axial strain of optical fiber sensors, achieving the shape perception of a medical robotic arm [17]. Dogu et al. studied a continuous equation-solving method based on the relationship model between strain–curvature–displacement and reconstructed the displacement field of beam structures according to the curvature value [18].

It is worth noting that the conventional curvature propagation method described above, which establishes the strain–curvature function relationship, is primarily applicable to flat-plate structures. However, the variable curvature of the wing leading edge, being a curved surface structure, results in changes in the arc angle during deformation. This variation leads to significant discrepancies between the curvature information calculated based on this function at measured points and the actual curvature. To address this issue, a strain–arc curvature function suitable for dynamically changing surface curvature angles of the wing leading edge is proposed based on the curvature radius of the leading-edge surface and measured strain, aiming to improve the accuracy of the curvature information for sensing points [19].

Conventional curvature propagation methods require linear interpolation to obtain curvature information for unattached sensor positions. However, the linear interpolation function, being a first-order linear function, accumulates errors between the interpolated curvature features and the actual curvature distribution, thereby affecting the accuracy of propagation reconstruction. Therefore, this study proposes a curvature propagation method based on node curvature vectors (NCV-CPM). By adding nodes within the segmented leading-edge arc sections, the aim is to enhance the order of curvature fitting functions. By constructing a least squares error function between theoretical curvature and actual curvature, node curvature vectors are solved to address the cumulative error problem. Additionally, by establishing a curvature–strain mapping function relationship, it is possible to achieve the inverse reconstruction of the leading-edge strain field based on the curvature distribution [20].

The higher the accuracy requirement for the morphing of the variable wing leading-edge morphological reconstruction, the greater the number of sensors needed, leading to a significant increase in the complexity of the monitoring system. To address this, researchers utilize optimization algorithms to obtain the optimal sensor layout, such as genetic algorithms [21], simulated annealing [22], and particle swarm optimization [23]. Genetic algorithms require determining suitable crossover and mutation rate parameters based on the size of the established sample library, while simulated annealing exhibits slow convergence and is influenced by multiple parameters to be optimized. In contrast, particle swarm optimization requires fewer optimization parameters and does not necessitate constructing a sample library. Therefore, this study proposes the adoption of particle swarm optimization to iteratively determine the optimal number of virtual sensing points, thereby simplifying the complexity of the sensor network.

The remainder of this study is organized as follows. Section 2 outlines the principles of the wing leading-edge morphological reconstruction method based on NCV-CPM. Section 3 describes the simulation model of the variable wing leading-edge structure and presents the simulation verification results of the NCV-CPM. Section 4 discusses the strain monitoring system for the variable wing leading-edge structure and presents the experimental verification results of the NCV-CPM. Section 5 concludes by summarizing the innovations and implementation outcomes of the NCV-CPM.

## 2. NCV-CPM Methodology

### 2.1. Construction of Strain–Arc Curvature Function for Sensing Points

**Figure 1:**
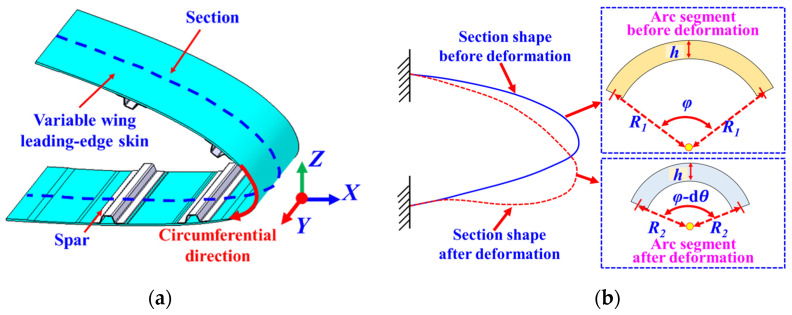
(**a**) Section selected in the leading edge of the variable wing; (**b**) comparison of the selected section before and after deformation.

Since the variable wing leading edge only undergoes bending deformation [24], and the morphological changes along the *Y*-direction remain consistent throughout the deformation process, it is sufficient to select a section along the circumferential direction of the wing leading edge as the research object, as illustrated in Figure 1a. In the selected circumferential section of the leading edge, any arbitrary arc segment can be chosen for analysis, as depicted in Figure 1b.

When the wing leading edge is undeformed, the curvature radius of the neutral layer within the selected arc segment is denoted as *R*_1_, and its corresponding arc angle is *φ*, with a skin thickness of *h*. With the internal actuation mechanism driving the bending deformation of the wing leading edge, the curvature radius of the neutral layer within the selected arc segment after deformation is defined as *R*_2_, with the corresponding arc angle being *φ*−d*θ*, while the skin thickness remains unchanged at *h*.

(1) $$\varepsilon =\frac{\left(R_{2}+\frac{h}{2}\right)\left(\varphi -d\theta \right)-\left(R_{1}+\frac{h}{2}\right)\varphi }{\left(R_{1}+\frac{h}{2}\right)\varphi }$$

Assuming that the arc length of the neutral surface within the selected arc segment remains constant during the deformation process [25]

(2) $$R_{2}\left(\phi -d\theta \right)=R_{1}\phi$$

Substituting Equation (2) into Equation (1), the curvature radius *R*_2_ of the neutral layer after deformation for the selected arc segment can be derived:

(3) $$R_{2}=\frac{hR_{1}}{2R_{1}\varepsilon +h\varepsilon +h}$$

Therefore, the surface curvature *q* of the selected arc segment after deformation can be expressed in terms of the measured strain *ε* at the measurement point, the skin thickness *h*, and the radius *R*_1_ of the neutral layer before deformation at the wing leading edge, as shown in Equation (4).

(4) $$q={\left(R_{2}+\frac{h}{2}\right)}^{-1}=\frac{h+2\varepsilon R_{1}}{\left(1+\varepsilon \right)hR_{1}}$$

Defining Equation (4) as the “Strain–Arc Curvature Function,” in subsequent validations of leading-edge morphing, the strain data from the surface-sensing measurement points on the leading-edge skin, along with the corresponding curvilinear angles of each arc segment on the wing leading edge, are inputted into this function model. This enables the precise calculation of curvature information at the discrete locations of the sensing points.

### 2.2. Reconstruction of Wing Leading-Edge Curvature and Strain Field Based on Node Curvature Vectors

**Figure 2:**
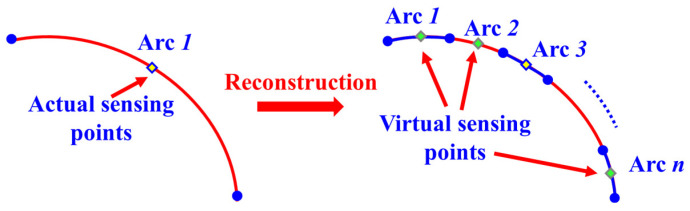
Schematic diagram of arc division density optimization.

Based on the curvature calculation method using discrete actual sensing points as described in Section 2.1, curvature information and strain information at positions where strain sensors are not attached cannot be obtained. Therefore, this section proposes a method for reconstructing the curvature field and strain field of the wing leading edge based on solving the curvature node vector of arc segments. This method can obtain curvature and strain distributions, corresponding to virtual sensing points on the wing’s leading edge—positions where actual sensors are not deployed—thereby achieving an equivalent expansion of curvature/strain sensing data. Based on this, it is possible to increase the density of segment partition without increasing the number of actual sensor measurement points, thereby improving the accuracy of morphological reconstruction, as shown in Figure 2.

**Figure 3:**
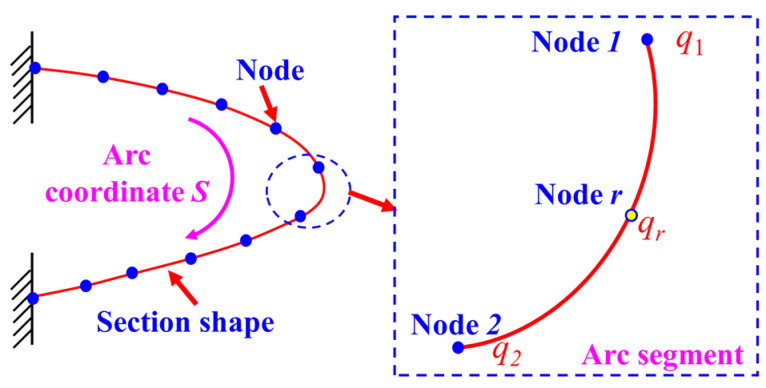
Arc segment node definition.

The core idea of the method is as follows: Firstly, according to the discrete layout scheme of actual measurement points, the wing leading-edge section is divided into a finite number of arc segments, as illustrated in Figure 2. To enhance the order of the curvature fitting function within each segment, an internal node *r* is added within each arc segment, except at the two ends [26], as depicted in Figure 3.

Next, the construction of the curvature node vector for each arc segment is undertaken. Taking the *m*th arc segment as an example, the curvature information from the two endpoints and one internal node of this segment is utilized to construct a curvature node vector, denoted as *q_Node_*, as shown in Equation (5).

(5) $$q_{Node}={\left[\begin{array}{ccc} q_{1} & q_{2} & q_{r} \end{array}\right]}^{T}$$

The theoretical curvature information at any point within the segment can be obtained by computing the curvature vector and curvature functions (*N*_1_, *N*_2_, *N*_r_), as illustrated in Equation (6). By substituting the arc length information s of the discrete measurement points into this equation, the theoretical curvature of the discrete measurement points can be calculated.

(6) $$q(s)=\sum_{i=1,2,r}N_{i}q_{Node},\;\left\{\begin{array}{l} N_{1}=2\xi^{2}-3\xi +1\\ N_{2}=2\xi^{2}-\xi \\ N_{r}=4\xi -4\xi^{2} \end{array}(\xi =s/L_{e}\in [0,\;1])\right.$$

where *s* represents the arc length parameter, and *L_e_* denotes the arc length of the segment.

Furthermore, Equation (6) is transformed into a matrix form that incorporates the curvature node vector *q_Node_* of the arc segment:

(7) $$q(q_{Node})=Cq_{Node},\;C=[\begin{array}{ccc} N_{1} & N_{2} & N_{r} \end{array}]$$

Based on the curvature *q_m_* calculated from Section 2.1 for the *m*th discrete actual measurement point, combined with the theoretical curvature of this point derived from the above analysis, a least squares error function model between the two is established, yielding the following [27]:

(8) $$\Phi_{e}(q_{Node})={\left\|q(q_{Node})-q_{m}\right\|}^{2}$$

By taking the derivative of the error function model concerning the curvature vector and setting it to zero, the curvature stiffness equation is obtained, as shown in Equation (9).

(9) $$\frac{\partial \Phi_{e}(q_{Node})}{q_{Node}}=k_{e}q_{Node}-f_{e}=0$$

where *k_e_* represents the curvature stiffness matrix, and *f_e_* denotes the curvature load matrix, as illustrated in Equation (10).

(10) $$\left\{\begin{array}{c} k_{e}=\sum_{i=1}^{n}\int_{\frac{(i-1)L_{e}}{n}}^{\frac{iL_{e}}{n}}(C^{T}C)dX\\ f_{e}=\sum_{i=1}^{n}\int_{\frac{(i-1)L_{e}}{n}}^{\frac{iL_{e}}{n}}(C^{T}q_{m})dX \end{array}\right.$$

Subsequently, based on the segmentation scheme of the arc segments, the curvature stiffness equations for each segment are assembled into the overall curvature stiffness equation. The solution of this equation provides the curvature vector at each node for every arc segment [28]. Combining Equation (6), further computations yield the curvature distribution information at locations on the wing’s leading-edge surface where sensors are not deployed (referred to as virtual sensing points).

**Figure 4:**
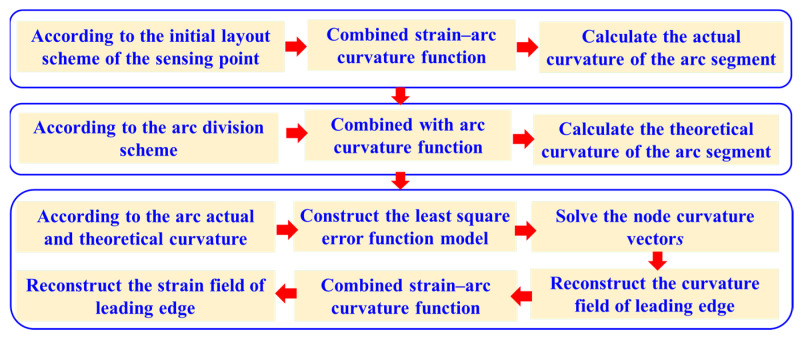
Curvature and strain field reconstruction process based on node curvature vectors.

It is noteworthy that, in conjunction with the curvature–strain mapping function relationship depicted in Equation (11), a reverse process can be employed to reconstruct the strain field at the leading edge. This, in turn, offers data support for structural health monitoring and condition assessment of the wing’s leading edge, as depicted in Figure 4.

(11) $$\varepsilon =\frac{(qR_{1}-1)h}{(2-qh)R_{1}}$$

### 2.3. Morphological Reconstruction Based on Curvature Propagation Method

The core idea of the wing leading-edge morphological reconstruction method based on the curvature propagation method is as follows. Firstly, following the segmentation scheme for the arc segments and leveraging principles of differential geometry, calculate the coordinate increments between the endpoints and starting points of all the arc segments. Secondly, starting from the initial segment, cumulatively add the coordinate increments of all arc segments. This process yields the coordinate positions after the deformation of the wing leading edge [29].

**Figure 5:**
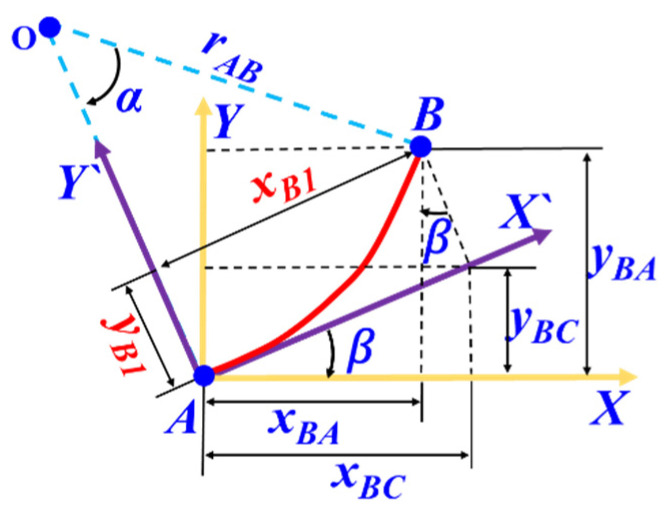
Coordinate increment calculation of arc segment.

Assuming the existence of the arc segment *AB*, as depicted in Figure 5, the principal coordinate system *XY* is established at the location of the arc segment, and the tangent line at starting point *A* is defined as the *X’* coordinate axis to establish the auxiliary coordinate system *X’Y’*. The projections of point *B* on the principal coordinate system *XY* are denoted as *x_BA_* and *y_BA_*, while the projections of point *B* on the auxiliary coordinate system *X’Y’* are denoted as *x_B_*_1_ and *y_B_*_1_. Furthermore, *x_BC_* and *y_BC_* represent the projection of *x_B_*_1_ onto the principal coordinate system *XY*.

According to geometric relationships, *x_BA_* and *y_BA_* can be expressed as follows:

(12) $$\left\{\begin{array}{c} x_{BA}=x_{B1}\times \cos\beta -y_{B1}\times \sin\beta \\ y_{BA}=x_{B1}\times \sin\beta +y_{B1}\times \cos\beta \end{array}\right.,\;\beta =\int_{0}^{s}q(s)ds$$

where *β* represents the angle between the tangent line at point *A* and the horizontal axis.

In the auxiliary coordinate system *X’Y’*, the expressions for *x_B_*_1_ and *y_B_*_1_ are given as follows:

(13) $$\begin{array}{cc} \left\{\begin{array}{l} x_{B1}=r_{AB}\times \sin a\\ y_{B1}=r_{AB}\times \left(1-\cos a\right) \end{array},\right. & \begin{array}{cc} a=\frac{S}{r_{AB}} & \;r_{AB}=\frac{q_{A}+q_{B}}{2q_{A}q_{B}} \end{array} \end{array}$$

where *α* represents the angle between the radii of points *A* and *B*, *r_AB_* denotes the average radius of the arc segment *AB*, *S* represents the size of the arc segment length, and *q_A_* and *q_B_*, respectively, represent the curvature at points *A* and *B*.

By combining Equations (12) and (13), the coordinate increments *x_BA_* and *y_BA_* relative to point *A* can be computed for point *B* on the arc segment. Cumulatively adding the coordinate increments of all arc segments allows for the reconstruction of the cross-sectional propagation of the wing leading edge after deformation.

### 2.4. Optimization of Virtual Sensing Point Quantity Based on Particle Swarm Algorithm

According to the particle swarm optimization algorithm, the sum of the virtual perception points and the actual measurement points in Section 2.2 is defined as the number of particles. By iteratively adjusting the number of particles, the optimal number of virtual perception points is sought, aiming to improve the accuracy of wing leading-edge propagation reconstruction without increasing the number of actual sensors. Considering the symmetry of the variable curvature wing leading-edge structure and the randomness of deformation, both actual and virtual perception points are arranged in an equidistant layout. The optimization process is as follows.

Firstly, based on the sensor layout initialization scheme, several actual strain sensors are uniformly distributed on the wing leading-edge surface. Combined with the method described in Section 2.2, approximate global measurements of the leading-edge surface curvature and strain distribution characteristics are obtained.

Secondly, utilizing the particle swarm optimization algorithm, along with Equation (14), the number of particles *n* is updated [30,31], yielding the following:

(14) $$\left\{\begin{array}{l} n\left(t+1\right)=n\left(t\right)+v\left(t+1\right)\\ v\left(t+1\right)=w\left(t\right)v\left(t\right)+C_{1}r_{1}\left(p_{g}\left(t\right)-n\left(t\right)\right) \end{array}\right.$$

where *t* represents the iteration count of particles, *n_i_*(*t*) denotes the number of measurement points at the *t*th iteration of particles, *n_i_*(*t*+1) represents the number of measurement points at the *t*+1th iteration of particles, *v_i_(t*+1) signifies the updating velocity at the *t*+1th iteration of particles, and *p_g_*(*t*) stands for the optimal number of measurement points obtained in the previous t iterations of particles. *C*_1_ is the weighting coefficient for particles tracking their own historical best value, typically set to 2, and *r*_1_ is a uniformly distributed random number within the interval [0, 1]. *w*(*t*) represents the updating velocity coefficient at the *t*th iteration of particles, as shown in Equation (15).

(15) $$w\left(t\right)=w_{\max}-iter\times \frac{w_{\max}-w_{\min}}{iter_{\max}}$$

where *iter*_max_ represents the maximum iteration count for the algorithm, and *iter* denotes the current iteration count. Typically, *w*_max_ is set to 0.9, and *w*_min_ is set to 0.4.

**Figure 6:**
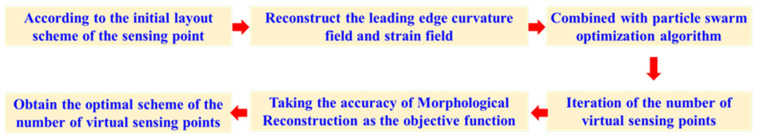
Number optimization of virtual sensing points based on particle swarm optimization algorithm.

Furthermore, following the method described in Section 2.2, curvature and strain information corresponding to different particle positions is obtained after each iteration. Building upon this information and combining it with the wing leading-edge propagation reconstruction method outlined in Section 2.3, the curvature accuracy is computed and compared for each iteration of particle positions. This process facilitates the determination of the optimal number of virtual perception points aimed at wing leading-edge propagation reconstruction, as depicted in Figure 6.

To evaluate the effectiveness of the proposed method for wing leading-edge propagation reconstruction, the Euclidean distance *d*(*i*) between each validation point on the wing leading edge and the coordinate origin is defined, as shown in Equation (16).

(16) $$d(i)=\sqrt{x{\left(i\right)}^{2}+y{\left(i\right)}^{2}}$$

where *d*(*i*) denotes the Euclidean distance between the *i*th validation point and the coordinate origin, *x*(*i*) represents the deformed *X*-coordinate of the *i*th validation point, and *y*(*i*) denotes the deformed *Y*-coordinate of the *i*th validation point.

Based on this, the absolute error, relative error, root mean square error, and mean relative error of the reconstruction for each validation point on the wing leading edge can be represented as follows:

(17) $$\left\{\begin{array}{ll} AE\left(i\right)=\left|d^{Method}\left(i\right)-d^{Ref}\left(i\right)\right|, & RE\left(i\right)=100\%\times \left|\frac{d^{Ref}\left(i\right)-d^{{\;}^{Method}}\left(i\right)}{d^{Ref}\left(i\right)}\right|\\ RMSE=\sqrt[{2}]{\frac{1}{m}\sum_{i=1}^{m}{\left(d^{{\;}^{Method}}\left(i\right)-d^{Ref}\left(i\right)\right)}^{2}}, & MRE=\frac{1}{n}\sum_{i=1}^{n}RE\left(i\right) \end{array}\right.$$

where *AE*(*i*) represents the absolute error of the morphological reconstruction for the *i*th validation point, *RMSE* stands for the root mean square error of the morphological reconstruction results for *m* validation points, *RE*(*i*) denotes the relative error of the morphological reconstruction for the *i*th validation point, *MRE* is the average of the relative errors of morphological reconstruction for *m* validation points, *d^Method^*(*i*) represents the reconstructed displacement for the *i*th validation point, and *d^Ref^*(*i*) is the true displacement for the *i*th validation point.

## 3. Simulation Verifications

### 3.1. Construction of Variable Wing Leading-Edge Model

**Figure 7:**
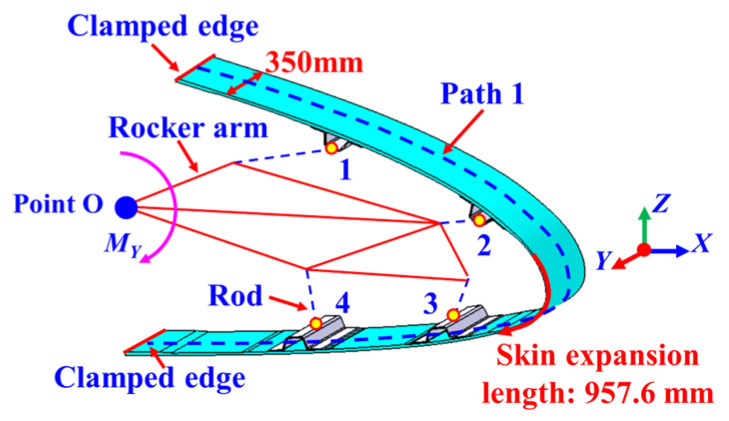
The leading edge of the morphing wing.

The swept-wing leading-edge structure was composed of skin, a spar, and an internally embedded leading-edge deformation drive mechanism [32], as illustrated in Figure 7. The chordwise direction of the leading-edge structure was defined as the *X*-direction, the vertical height direction of the structure was defined as the *Z*-direction, and the lateral direction of the skin was defined as the *Y*-direction. In the simulation model of the wing leading edge, the unfolded length of the leading-edge skin was 957.6 mm, with a width of 350 mm and a thickness of 3 mm.

The internal driving mechanism of the variable leading edge comprised a rocker arm and four connecting rods. The leftmost endpoint of the rocker’s arm was hinged at the loading point O. One end of each of the four connecting rods was connected to the rocker’s arm through hinge points 1, 2, 3, and 4, respectively, while the other end was connected to the four spars through brackets. In the finite element simulation analysis, the root of the leading edge was set as a fixed support. By applying a bending moment *M_Y_* at the loading point O, the rocker’s arm drove the four connecting rods to deflect, thereby achieving control over the deformation of the wing’s leading edge.

Under the action of the leading-edge deformation drive mechanism, the wing leading edge can rotate clockwise around the fixed point O. During the rotation process, the aerodynamic performance of the aircraft, including lift, drag, and stability, will correspondingly change. This design facilitates maintaining stable flight performance to the maximum extent possible during different mission modes.

A simulation validation of the morphological reconstruction method for the skin structure of the variable wing leading edge was conducted for four different leading-edge deformation states, with deflection angles of 2°, 4°, 8°, and 10°, respectively.

### 3.2. Results and Discussion

**Figure 8:**
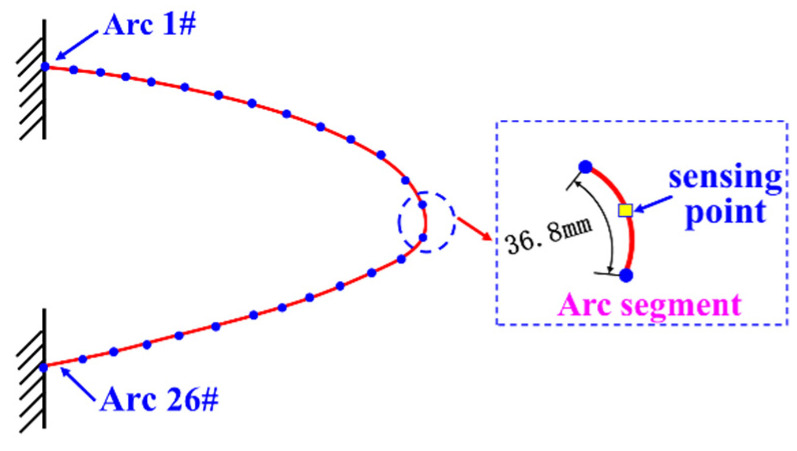
Arc segment division scheme.

Due to the predominant bending deformation of the leading-edge structure, the strain and displacement distributions on the skin surface remained relatively constant along the *Y*-direction. In subsequent simulation validations, we found that it was only necessary to extract the strain data along the surface Path1 of the skin. Path1, located on the leading-edge circumferential section, was divided into 26 equally spaced arc segments. At the center of each segment, one strain-sensing point was positioned, and the nodes at both ends of each segment served as validation points for assessing the reconstruction effectiveness of the strain field, curvature field, and displacement field, as illustrated in Figure 8.

#### 3.2.1. Comparison of Curvature and Strain Reconstruction Errors between LI-CPM and NCV-CPM

**Figure 9:**
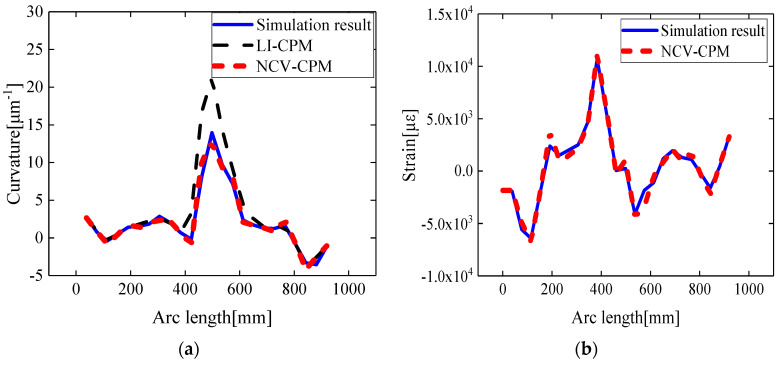
Comparison of reconstruction results in Path1: (**a**) curvature result; (**b**) strain result.

Taking the example of a leading-edge deflection angle of 10°, simulation data corresponding to 26 strain-sensing points along Path1 were extracted according to the method described in Section 2.2. The reconstructed surface curvature field and strain field information of the variable wing leading edge is depicted in Figure 9.

According to Figure 9a, along the circumferential direction of the wing leading edge, the curvature angles decreased and then increased, with the minimum curvature angle being observed in the central region. Therefore, after the deformation of the variable wing leading edge, the reconstructed curvature extremum was located in the middle region of the leading edge. Additionally, as indicated in Figure 9b, due to the predominant control of the wing leading edge by the main spar, the reconstructed strain field exhibited a sudden change at the main spar location.

The curvature and strain reconstruction results for various validation points along Path1 are illustrated in Figure 9. For the conventional curvature propagation algorithm based on linear interpolation (LI-CPM), the relative error average of the curvature reconstruction was 20.24%, and the root mean square error was 2.61 μm^−1^. In contrast, the curvature propagation algorithm based on segment curvature nodal vectors (NCV-CPM) achieved a reduced relative error average of 5.98% and a decreased root mean square error of 0.42 μm^−1^, while the relative error average for the strain reconstruction was 4.66%.

There are two main reasons for this phenomenon: Firstly, the curvature calculation accuracy of the sensing points is improved due to the strain–curvature function model proposed in Section 2.1. Secondly, the adoption of higher-order curvature functions to solve the curvature vector at element nodes enhances the accuracy of curvature calculation at locations where actual sensors are not deployed.

Therefore, this method, utilizing a small number of discrete strain-sensing points, not only significantly improved the curvature reconstruction accuracy at the positions of unattached sensors (virtual sensing points) but also allowed for the reverse reconstruction of the leading-edge strain distribution information.

#### 3.2.2. Optimization of Virtual Sensing Node Quantities Based on Particle Swarm Algorithms

**Figure 10:**
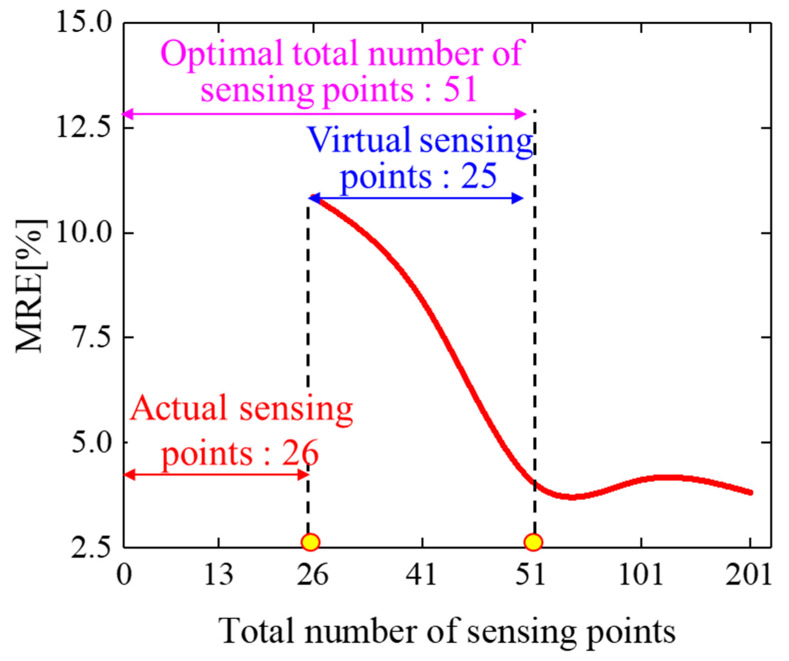
The number optimization process of sensing points based on the particle swarm optimization algorithm.

Taking the example of a leading-edge deflection angle of 10°, the entire surface strain/curvature distribution data for the leading edge were reconstructed based on the simulated data from 26 strain-sensing points, as described in Section 3.2. Furthermore, utilizing the particle swarm optimization algorithm outlined in Section 2.4, the reconstruction accuracy of the leading-edge morphology was calculated after each iteration of particle quantity. This was carried out to determine the optimal number of virtual sensing points required for the morphological reconstruction of the wing leading edge, as illustrated in Figure 10.

During the particle quantity iteration process, the root mean square error of the morphological reconstruction of the variable wing leading edge, starting from the initial 26 actual sensing points, decreased from 28.84 mm to 7.06 mm.

As depicted in Figure 10, with the continuous increase in the number of virtual sensing points, the reconstruction error of the variable wing leading-edge morphology steadily decreased. When the sum of the virtual and actual sensing points exceeded 51, the average relative error of the leading-edge morphology reconstruction converged and no longer decreased. Therefore, uniformly distributing 51 sensing points along the circumferential direction of the wing leading edge could be considered the optimal sensor layout scheme. In this scheme, 26 strain data points were acquired from actual sensors, while the additional 25 strain data points from virtual sensing points could be calculated using the reconstruction method described in Section 2.2.

#### 3.2.3. Comparison of Morphology Reconstruction Errors between LI-CPM and NCV-CPM

To assess the feasibility of the aforementioned reconstruction method, simulations were conducted to verify the effectiveness of enhancing the accuracy of the morphological reconstruction of the variable wing leading edge based on the curvature nodal vector solution and point optimization results.

**Figure 11:**
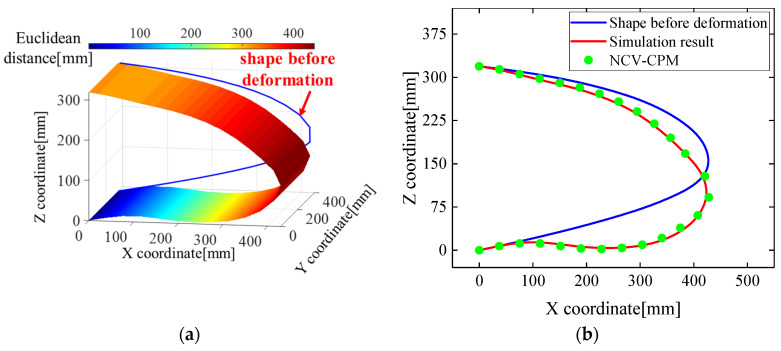
Morphological reconstruction results of NCV-CPM for wing leading edge of deflection angle 10°: (**a**) Euclidean distance cloud map; (**b**) reconstruction effect comparison.

At a leading-edge deflection angle of 10°, the reconstructed morphology curve of the wing leading edge along Path1 closely aligned with the simulated displacement curve, as illustrated in Figure 11.

**Figure 12:**
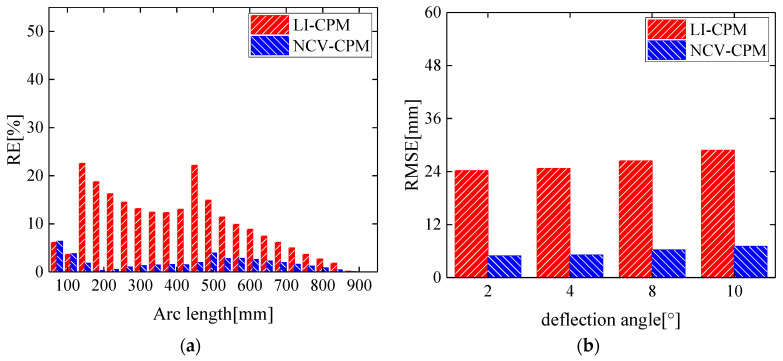
Propagation reconstruction effect comparison of LI-CPM and NCV-CPM: (**a**) relative error under 10° deflection angle of leading edge; (**b**) root mean square error under four leading edge deflection angles.

The conventional curvature propagation method (LI-CPM) based on linear interpolation yielded an average relative error and root mean square error of morphological reconstruction of 10.85% and 28.84 mm, respectively. In contrast, the proposed morphological reconstruction method (NCV-CPM) in this study achieved an average relative error and root mean square error of 3.34% and 7.06 mm, respectively, as depicted in Figure 12a.

A comparison of the morphological reconstruction accuracy between the conventional curvature propagation method and the algorithm proposed in this study is presented in Figure 12b for leading-edge deflection angles of 2°, 4°, 8°, and 10°.

From Figure 12, it is evident that, compared to the conventional curvature propagation method based on linear interpolation, the method proposed in this study significantly improved the morphological reconstruction accuracy at four different deflection angles. This improvement stemmed from the fact that the conventional curvature propagation method utilized a first-order linear interpolation function to obtain curvature information, which failed to meet the requirements for fitting the leading-edge curvature field, thereby affecting the accuracy of the morphological reconstruction. In contrast, the method proposed in this study enhanced the reconstruction effectiveness of the curvature fitting function by introducing additional nodes within the segments, thereby avoiding the influence of calculation errors in curvature information due to unattached sensor positions on the accuracy of the wing leading-edge morphology reconstruction.

Furthermore, this study employed a particle swarm algorithm to determine the optimal number of virtual sensing points required for the morphological reconstruction of the wing leading edge, thereby reducing the complexity of the sensor network.

## 4. Experimental Validations

### 4.1. Construction of Monitoring System for Variable Wing Leading-Edge Morphology

**Figure 13:**
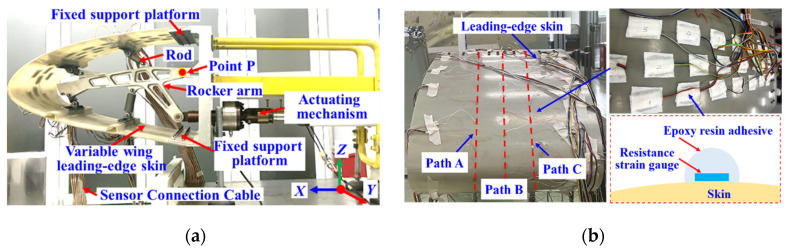
(**a**) Experimental system of wing leading-edge deformation reconstruction; (**b**) integration between strain sensor and leading edge.

The variable wing leading-edge monitoring experimental system primarily consisted of a dynamic strain acquisition instrument, resistance strain gauges, a fixed platform, laser displacement sensors, the variable wing leading edge, and its actuating mechanism, as depicted in Figure 13a.

The skin of the variable wing leading edge in the monitoring system is fabricated using an epoxy resin matrix reinforced with SW100A/6511 woven glass-fiber prepreg. The specific material parameters are detailed in Table 1.

The root of the wing’s leading edge was mounted on a fixed support platform, and when the rocker’s arm rotated around loading point P, four connecting rods drove the main spar to achieve precise control of the leading-edge deformation.

Experimental validation of the variable wing leading-edge morphological reconstruction method was conducted for four leading-edge deformation states with deflection angles of 3°, 6°, 9°, and 12°, respectively. The strain responses measured by strain gauges attached to the leading-edge skin surface were utilized as inputs for the morphological reconstruction method, as shown in Figure 13b.

To enhance the density of strain response information collection caused by the deformation of the wing leading edge, three complementary and staggered layouts of sensing points were designed along the chordwise direction of the wing. Combining the optimization results of the sensing point quantity in Section 3.2.2, three strain-sensing paths, namely, Path A, Path B, and Path C, were established on the surface of the wing leading-edge skin. Each path was equipped with 17 strain-sensing points arranged at equal intervals, with a spacing of 57.5 mm between adjacent points.

**Figure 14:**
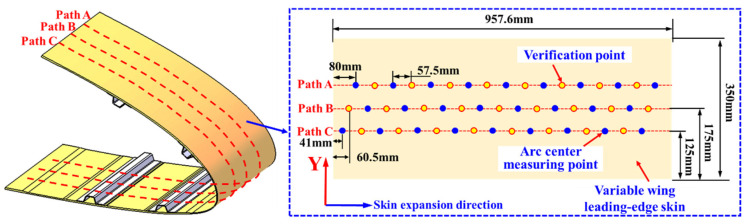
Layout scheme of strain-sensing measuring points in staggered complementary mode.

Among these 51 sensing points, 26 points (highlighted in yellow) were selected to gather data as inputs for the morphological reconstruction method proposed in this study, while the remaining 25 points (highlighted in blue) were used for evaluating the curvature/strain reconstruction effectiveness, as illustrated in Figure 14.

### 4.2. Results and Discussion

#### 4.2.1. Comparison of Curvature and Strain Reconstruction Errors between LI-CPM and NCV-CPM

**Figure 15:**
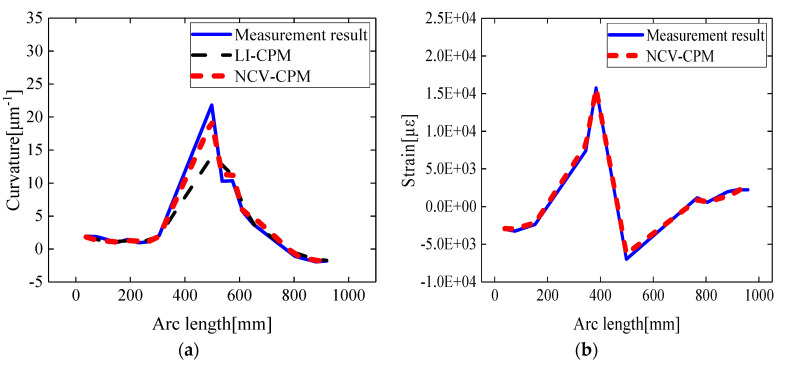
Comparison of reconstruction results in Path B: (**a**) curvature results; (**b**) strain results.

Taking the leading-edge deflection angle of 12° as an example, based on the method presented in Section 2.2, the reconstructed skin curvature field and strain field after unfolding the variable wing leading edge were obtained. These distributions are depicted in Figure 15a,b. The extremum of the reconstructed curvature distribution was located at an arc length of 500 mm, while the reconstructed strain field exhibited a sudden change in the position of the main spar. Both phenomena were consistent with the results obtained from numerical simulations.

Compared to the conventional curvature recursion method (LI-CPM), the average relative error of the curvature reconstruction for the validation points on Path B of the wing leading edge decreased from 25.09% to 6.32%, and the root mean square error decreased from 2.15 μm^−1^ to 0.81 μm^−1^, as shown in Figure 15a.

The experimental results demonstrated that the conventional curvature recursion method, due to neglecting the initial curvature and sole reliance on linear interpolation to obtain curvature information for unattached sensor positions, resulted in accumulated errors between the calculated curvature field distribution and the actual curvature distribution. In contrast, the proposed method in this study, by sequentially constructing a strain–curvature function that could characterize the dynamic variation in the curvature of the variable leading edge and solving the curvature node vectors of the arc segments, effectively mitigated the impact of the aforementioned cumulative errors on the accuracy of the curvature field reconstruction.

As depicted in Figure 15b, this method, based on the curvature–strain mapping function and the characteristics of the reconstructed curvature field distribution, could also achieve the inverse reconstruction of the strain field of the wing leading edge. This provided data support for the structural health monitoring and fatigue prediction of the wing leading edge.

#### 4.2.2. Comparison of Morphology Reconstruction Errors between LI-CPM and NCV-CPM

**Figure 16:**
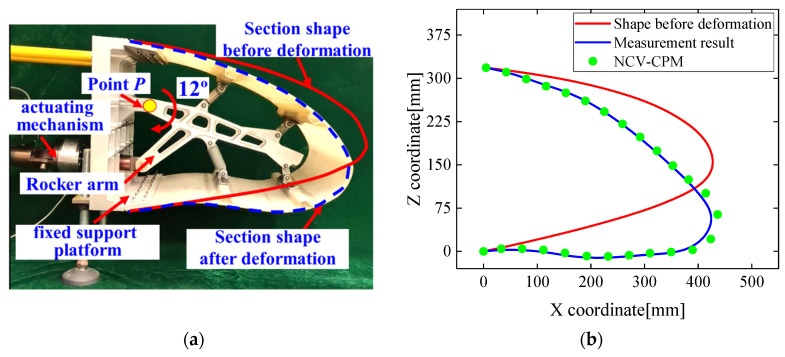
Morphological reconstruction results of NCV-CPM for wing leading edge of deflection at angle of 12°: (**a**) leading-edge deflection diagram; (**b**) reconstruction effect comparison.

The actual form of the leading edge was evaluated using measurements from laser displacement sensors to assess the effectiveness of the method proposed in this study for morphological reconstruction. Taking a leading-edge deflection angle of 12° as an example, the reconstructed variable wing leading-edge cross-sectional form closely matched the actual form curve, as illustrated in Figure 16.

**Figure 17:**
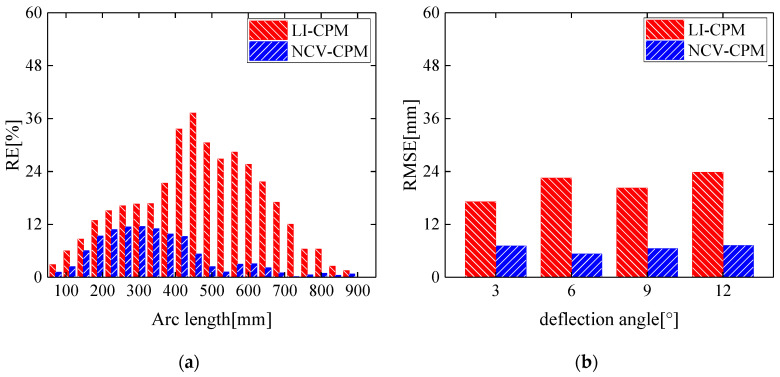
Propagation reconstruction effect comparison of LI-CPM and NCV-CPM: (**a**) relative error under 12° deflection angle of leading edge; (**b**) root mean square error under four leading-edge deflection angles.

As depicted in Figure 17a, when the leading-edge deflection angle was 12°, the morphological reconstruction error of the wing leading edge using NCV-CPM was significantly lower than that of the conventional LI-CPM, with the average relative error of morphological reconstruction decreasing from 17.05% to 4.92%. For deformation states with leading-edge deflection angles of 3°, 6°, and 10°, the morphological reconstruction accuracy of the method proposed in this study was notably superior to that of the conventional curvature recursion method, as shown in Figure 17b.

The experimental results demonstrate that the proposed method achieves an improvement in morphological reconstruction accuracy by reducing both the curvature calculation error at sensing points and the cumulative error in curvature reconstruction.

## 5. Conclusions

To meet the self-perception requirements of the variable wing leading-edge morphology based on biomimetic principles, a morphological reconstruction method for the variable wing leading edge was proposed in this study, based on the relationship model between the strain and surface curvature function and the solution of segment node curvature vectors. The main work carried out is summarized as follows:

(1) The surface angle was introduced into the relationship model between strain and surface curvature to improve the accuracy of the curvature calculation for sensing points on the wing leading edge.
(2) A method for solving node curvature vectors based on high-order curvature fitting functions was proposed. Compared with the conventional curvature recursion method based on first-order interpolation functions, the error in curvature field reconstruction could be reduced from 25.09% to 6.32%. Furthermore, after obtaining the curvature field of the wing leading edge, its application in the strain–surface curvature function enabled the inverse reconstruction of the strain field of the wing leading edge.
(3) An optimization method for the number of sensing points based on the particle swarm algorithm was proposed, determining the optimal number of virtual sensing points. This not only simplified the complexity of the wing leading-edge sensing network but also improved the accuracy of morphological reconstruction. The results showed that, compared with the conventional curvature recursion method, the morphological reconstruction error of the wing leading edge decreased from 17.05% to 4.92%.

The proposed method was unaffected by modeling accuracy and did not require consideration of structural material parameters and external load information. It could reconstruct approximate global measured information of the variable wing leading-edge curvature field, strain field, and displacement distribution, thereby providing real-time data support for the adaptive closed-loop control and structural health monitoring of variable wings based on biomimetic principles.

## Figures and Tables

**Table 1:** Material parameters of the glass-fiber prepreg.

E_1_ (GPa)	E_1_ (GPa)	Nu_12_	G_12_ (GPa)	ε_t_ (μ)	ε_c_ (μ)
47.7	13.3	0.12	47.5	33,166	13,538

## Data Availability

The data presented in this study are available upon request from the corresponding author. The data are not publicly available due to privacy.
